# MFAP2 promotes metastasis and drug resistance by regulating epithelial-to-mesenchymal transition through EGFR signaling pathway in colorectal cancer cells

**DOI:** 10.1016/j.gendis.2025.101800

**Published:** 2025-08-12

**Authors:** Zhicheng He, Yuanzhi Chen, Shuting Yang, Cheng Chen, Yingying He, Shubai Liu

**Affiliations:** a State Key Laboratory of Phytochemistry and Natural Medicines, Kunming Institute of Botany, Chinese Academy of Sciences, Kunming, Yunnan 650201, China; b University of Chinese Academy of Sciences, Beijing 100049, China; c School of Chemical Science & Technology, Yunnan University, Kunming, Yunnan 650091, China

**Keywords:** Colorectal cancer, Epithelial-to-mesenchymal transition, Metastasis, MFAP2, Therapeutic target, Virtual screening

## Abstract

Colorectal cancer (CRC) is a prevalent condition, with metastasis spread as the primary cause of mortality. However, MFAP2 function in CRC progression and its regulatory mechanisms in metastasis remain poorly understood. To investigate the status of MFAP2, an extensive analysis was conducted using multiple clinical databases and transcriptomic data from CRC metastasis patients’ tissues and several CRC cell lines. The efficacy of standard first-line chemotherapy drugs (5-fluorouracil, irinotecan, and oxaliplatin) were evaluated for any potential drug resistance. Virtual screening and molecular docking were used to identify potential inhibitory compounds that could be effective against CRC. Moreover, MFAP2 expression was found to be significantly higher in CMS4 CRC patients compared to those with other subtypes. This elevation in MFAP2 correlated with both tumor stromal score and tumor purity. Reducing MFAP2 expression led to a significantly decline in the functional capabilities of CRC cells and heightened their sensitivity to standard chemotherapy treatments. Results have identified MFAP2 as a key regulator in the metastasis of CRC, influencing processes like epithelial-to-mesenchymal transition through the EGFR-AKT-STAT3 signaling pathway. Therefore, MFAP2 emerges as a promising therapeutic target for anti-tumor efforts. Notable, three compounds were discovered that effectively bind and down-regulate MFAP2, which significantly impairs tumor cells migration. These findings revealed new functions of MFAP2, suggesting it plays a vital role in driving epithelial-to-mesenchymal transition, metastasis, and chemotherapy resistance in CRC. This provides a fresh perspective for developing treatment strategies. Overall, targeting MFAP2 may offer a more effective therapeutic option for CRC patients with CMS4.

## Introduction

Colorectal cancer (CRC) represents a prevalent global health challenge due to its high incidence and mortality rate.^1^ Metastasis is a key contributor to the deaths of newly diagnosed CRC patients.^2^^,^^3^ Therefore, it is essential to understand the progression of CRC and explore new therapeutic strategies. One vital process in CRC development is the epithelial-to-mesenchymal transition (EMT), where epithelial cells acquire mesenchymal properties.^4^^,^^5^ EMT plays a significant role in the tumor metastasis, particularly in CRC, and contributes to the challenges of resistance to chemotherapy and immunotherapy by enabling tumor cells to engage with supporting tumor-associated stromal cells that promote tumor growth.^6^^,^^7^ EMT is influenced by various regulators within multiple cell signaling pathway.^8^ Recent findings suggest that EMT may provide new prognostic markers or therapeutic targets for CRC treatment.^9^, ^10^, ^11^ Therefore, discovering the factors that govern EMT is crucial for advancing effective therapeutic strategies.

Microfibrillar-associated protein 2 (MFAP2), which is also referred as microfibril-associated glycoprotein 1 (MAGP1), has been extensively studied for its role in binding to various extracellular components such as fibrillin, collagen VI, tropoelastin, decorin, and biglycan.^12^ Additionally, MFAP2 has also been found to enhance the expression of genes associated with cell adhesion, motility, and matrix remodeling when act in cells.^13^, ^14^, ^15^ MFAP2 was identified as a valuable prognostic marker and a potential target for therapies in multiple cancers, particularly gastric cancer,^16^, ^17^, ^18^, ^19^, ^20^ hepatocellular carcinoma,^21^^,^^22^ melanoma,^23^ and breast cancer.^24^

In the context of colorectal cancer (CRC), MFAP2 plays an important role in the remodeling of extracellular matrix remodeling and tumorigenesis.^25^ Its expression is linked to the progression of colon adenocarcinoma^26^ and promoting CRC invasiveness.^27^ In addition, our earlier study found that MFAP2 was up-regulated in patients exhibiting EMT phenotype and those classified within consensus molecular subtype 4 (CMS4).^28^

Furthermore, a significant hurdle in the treatment of CRC is the resistance of cancer cells to chemotherapeutic drug, which often leads to diminished treatment efficacy and poorer prognosis. This resistance can stem from various mechanisms, including the activation of DNA damage responses, EMT processes, alterations in gene expression, and the inhibition of drug transport or target signaling pathways. Notably, MFAP2 has been linked to drug resistance in gastric cancer.^29^ However, the precise mechanism through which MFAP2 influences tumor metastasis and drug resistance remains inadequately understood. Therefore, it is essential to conduct further studies into the interaction between MFAP2, EMT, and tumor metastasis, as this could provide new insight into targeting EMT for CRC precise treatment.

In this study, multiple research methods were employed to investigate the potential mechanism of MFAP2 regulation in CRC tissues, tumor-adjacent tissues, and various CRC cell lines. The role of MFAP2 in promoting CRC progression was examined in stable knockdown cell lines. The clinical first-line chemotherapy drugs (5-fluorouracil, irinotecan, and oxaliplatin) for CRC were applied to evaluate the synergic effect of MFAP2 on drug resistance. Transcriptomic profile analysis was used to uncover the critical pathways and functions influenced by MFAP2 through integrated analysis. Additionally, the correlation analysis among MFAP2, biglycan (BGN), and thrombospondin 2 (THBS2) was explored with tumor scores and tumor purity. This was achieved through quantitative immunohistochemical analysis and Clinical Proteomic Tumor Analysis Consortium (CPTAC) transcriptomic/proteomic profiling data from CRC patients' tissues. Moreover, virtual screening and molecular docking, which play a crucial role in identifying new drug targets and rapidly assessing the potential efficacy of numerous compounds for targeted anti-tumor drugs,^30^, ^31^, ^32^ have been used to evaluate MFAP2 as a promising therapeutic target with potential clinical application.

## Materials and methods

### MFAP2 expression profiles analysis

Multiple datasets were employed to explore the potential prognostic value of MFAP2. Firstly, the TCGA transcriptome and CPTAC proteome data in UALCAN (http://ualcan.path.uab.edu) was used to analyze the expression difference of MFAP2 in normal colorectal tissues and CRC tissues. Secondly, GEPIA (http://gepia.cancer-pku.cn) was used to further validate and access with the expression level of MFAP2 in CRC in patients at various stages. Lastly, the expression difference of MFAP2 in disease-free and recurrent cases as well as the impact of MFAP2 expression level on disease-free survival, were examined using the CANCERTOOL (http://web.bioinformatics.cicbiogune.es/CANCERTOOL/datasetsInfo.php).

### Immunohistochemical staining

The colon cancer tissue microarrays were purchased from Shanghai Outdo Biotech Co., Ltd. to detect the expression level of MFAP2 (tissue samples from the National Human Genetic Resources Sharing Service Platform; serial number: 2005DKA21300). The formalin-fixed and paraffin-embedded patient's tissues were cut into at 4 μm-thick sections. One slide contains 160 colon tissue sections, 80 of which are tumor tissues and 80 of which are adjacent tissues. After being deparaffinized in xylene and rehydrated using a graded series of ethanol solutions, the slide was put into an oven set to 60 °C for 1 h. The sections were incubated with an antibody against MFAP2 (1:200; Solarbio, China) at 4 °C overnight following the 10-min inhibition of endogenous peroxidase by 3% hydrogen peroxide and the recovery of the antigen. According to the instructions of the BOSTER SABC detection protocol, sections were subsequently incubated with secondary antibodies conjugated with horseradish peroxidase (BOSTER, China). Using Image J (version 1.53), the positive staining rate was calculated as the expression level in order to evaluate of the variation in MFAP2 protein expression level between the tumor and adjacent tissues.

### Transcriptome profiling analysis

RNA was extracted from HCT116 shRNA control and MFAP2 knockdown cell lines using Trizol reagent (Invitrogen, Carlsbad, California, USA). RNA concentration and purity were determined by Nanodrop2000 (Thermo Fisher Scientific, Waltham, Massachusetts, USA), while RNA integrity was evaluated by agarose gel electrophoresis and RNA integrity number (RIN) value was determined by Agilent 2100 (Agilent Technologies Inc., Santa Clara, California, USA). The mRNA was isolated from total RNA Using Oligo(dT) magnetic beads through poly-A tail hybridization for transcriptomic analysis. Following fragmentation buffer treatment, RNA fragments of approximately 300 bp were isolated by magnetic bead screening. One-strand cDNA synthesis was performed using mRNA as a template with six-base random primers (random hexamers) under reverse transcriptase, followed by second-strand synthesis to form a stable double-strand structure. The double-stranded cDNA was processed using End Repair Mix to generate blunt ends, followed by addition of an “A” base at the 3′ end to connect the Y-shaped adapter. Libraries were sequenced on Illumina Novaseq 6000 Platform. Significantly Differentiated expression genes were identified using significance analysis of microarrays (SAM) with the R package “samr” (*P*_adj_ value < 0.05), and differential expression genes in the shMFAP2 group were enrichment analyzed by “R project” to study which biological processes these genes were involved in. Gene Set Enrichment Analysis (GSEA) was applied to identify enrichment functions of differentiated genes.

### Cell culture and plasmid transfection

HCT116 was chosen by western blotting analysis in four cell lines to investigate the impact of MFAP2 on colon cancer *in vitro* (Fig. S1). The construction of short-hairpin RNA (shRNA) targeting MFAP2 (sh-MFAP2) and control shRNA (mock, sh-Ctrl) was designed and synthesized by GeneChem (Shanghai, China; Table S1). The shRNAs were transfected in HCT116 cells according to the manufacturer's protocol of Lipofectamine 2000 (Invitrogen). After two weeks of selecting stably transfected cells in media containing with 0.8 μg/mL puromycin (Sigma–Aldrich Co.), and the western blotting analysis was used to confirm that MFAP2 was knockdown in HCT116 cells.

### Western blotting

Well growth cells cultured in six-well plates were washed twice with ice-cold phosphate-buffered saline solution (PBS), and then 300 μL of cellular lysates (RIPA: PMSF, 100:1) was added to each well. The total protein concentration was determined by NanoDrop (Thermo Fisher Scientific, USA). Equal amounts (30–50 μg) of whole cell lysates were loaded on a 10% SDS-PAGE gel with 80 V for 30 min and then 120 V 1.5 h for proteins separation. The proteins were electrophoretically transferred onto polyvinylidene fluoride membranes (100 V, 90–120 min). Following blocking with 5% non-fat milk in PBS with 0.02% Tween 20 detergent (PBST) at room temperature for 2 h, the membranes were incubated with primary antibodies, including MFAP2 (Solarbio, China), GAPDH (BBI Co., Ltd., China), epidermal growth factor receptor (EGFR; Proteintech, China), protein kinase B (AKT) (Proteintech), signal transducer and activator of transcription 3 (STAT3) (Proteintech), and vascular endothelial growth factor A (VEGFA) (Proteintech), p-EGFR (Cell Signaling Technology, USA), p-STAT3 (Cell Signaling Technology), and p-AKT^ser473^(Cell Signaling Technology) antibodies, at 4 °C overnight. Horseradish peroxidase-labeled goat anti-rabbit IgG and horseradish peroxidase-labeled goat anti-mouse IgG (BBI Co., Ltd., China) were used for detecting MFAP2 and GAPDH, respectively, after the secondary antibodies were incubated at 37 °C for 2 h. Finally, PVDF membranes were evenly coated with ECL luminescent solution. The intensity of protein bands was detected by Tanon photography and quantitatively analyzed by Image J.

### Cell proliferation and colony formation assay

For the CCK8 assay, 2500 cells (100 μL) per well were seeded into 96-well plates and cultured for 0, 1, 2, and 3 days. Each plate was then filled with 10 μL of cell counting kit-8 (CCK8) solution (Proteintech, China) and it was incubated for 2 h at 37 °C. A microplate reader (BioTek Instruments, Winooski, VT, USA) was used to measure the absorbance at 450 nm.

Six-well plates were seeded with 500 cells per well and the cells were cultured for 14 days in order to perform colony formation tests. Depending on the culture conditions the medium was changed every two or three days. Clones were then fixed with 4% paraformaldehyde and stained with 0.1% crystal violet. Light microscopy (Leica DM Microscope, Germany) was used to count colonies containing over 50 cells. Three duplicates of each experiments were performed.

### 3D tumor sphere assay

Tumor sphere assay was performed by mixing a single-cell suspension with extracellular matrix (BD Biosciences Matrigel catalog #356234) on ice, plated in triplicate into 12-well culture plates, and incubated in an incubator set at 37 °C with 5% CO_2_. Photographs of tumor spheres were taken on days 1, 3, 5, 7, and 10.

### Cell migration and invasion assay

Cell migration and invasion were examined by Transwell assay using 24-well Transwell chambers with an 8-mm pore size and a track-etched membrane (Corning, New York, USA). Cells (5–6 × 10^4^ cells/well in 100 μL) were plated into the upper chamber of 8-mm-pore-size Transwell chambers (Corning, New York, USA) for the migration assay. The lower chamber was filled with RPMI 1640 medium that containing 10% fetal bovine serum. After that, the chambers were incubated for 24 h at 37 °C. After removing the cells from the upper chamber, 0.1% crystal violet dye was used to count the membranes’ bottom surface. Matrigel (BD Biosciences Matrigel) was used in the Transwell chambers for the invasion assay. The migration assay was the same as other experimental procedures. Cell migration and invasion were quantified by counting five random fields under a microscope.

### MFAP2 knockdown impact on drug resistance

Firstly, sh-Ctrl cells were used to measure the IC_50_ values of three chemotherapeutic drugs (5-fluorouracil, irinotecan, and oxaliplatin). After that, for 48 h incubation period, the dosage concentration was set to IC_50_/2, IC_50_, and 2 IC_50_. Lastly, each well received 10 μL of cell counting kit-8 (CCK8) solution (Proteintech, China), which was incubated for 2 h at 37 °C. A microplate reader (BioTek Instruments, Winooski, Virginia, USA) was used to measure the absorbance at 450 nm.

### Transcriptomic and proteomic profiling

CPTAC proteomic and CANCERTOOL were used to extract the transcriptomic and proteomic profile data. It was accessed to the variations of MFAP2 expression in EMT, epithelial cells, and hypermutated patients, as well as in CMS1, CMS2, CMS3, and CMS4 patients. In addition, a correlation analysis was conducted between MFAP2 and EMT-related proteins was performed, including E-cadherin, N-cadherin, and vimentin.

### Immunofluorescence staining

After placing the glass covers in 6-well plates and giving them two PBS washes, the cells of the logarithmic growth stage were injected at a density of 50%–60% and cultivated for 48 h in a cell incubator. After discarding the medium, cells underwent three PBS washes and a 10-min fixation with 4% paraformaldehyde-PBS (1 mL/well). The cells were then infiltrated with 1 mL PBST plus 0.5% NP-40 (BBI Co., Ltd., China) on ice for 15 min after being washed three times with PBST. Following the completion of permeability, the cells were incubated with a 1.5% bovine serum albumin (BSA)/PBST solution for 30 min at room temperature after being rinsed three times with PBS-T. Vimentin (Proteintech, China), N-cadherin, and E-cadherin primary antibodies were diluted 1:100 in BSA/PBST solution and incubated at 4 °C for the entire night. Following primary antibody incubation, cells were rinsed three times with PBST (10 min each). The red fluorescent (*λ* = 594 nm) secondary antibody solution (Proteintech, China) was diluted (1:100) and treated with cells for 1 h at 4 °C in the dark. Following incubation, cells were rinsed three times in the dark for 5 min each using PBST. Following the addition of the proper quantity of anti-quench agent, the cover slide was inverted, sealed with nail oil, and examined and captured on camera using a fluorescence microscope (ThermoFisher Scientific, USA).

### MFAP2 analysis with tumor purity and tumor score

The differences between EMT, epithelial cells, and hypermutated patients in terms of stromal score, immune score, ESTIMATE score, and tumor purity were analyzed. Additionally, the correlations among MFAP2 and these indicators were explored, as well as the discrepancies between these indicators in CMS1∼CMS4 patients. Proteomic data from CPTAC served as the basis for aforementioned analyses.

### Correlation between MFAP2 and BGN and THBS2 expression in clinical tumor tissues

Three colon cancer tissue arrays immunohistochemical staining results were integrated (whole tissue assay was shown in Fig. S4). The BGN and THBS2 data were from our earlier study.^28^ It was analyzed the correlation between MFAP2 and BGN (*n* = 65 paired tissues) or THBS2 (*n* = 64 paired tissues). Meanwhile, CPTAC proteomic data and CANCERTOOL data provided additional validation for these correlation results between them.

### Virtual screening and molecular docking

The structure of the small-molecule compounds was downloaded from Drugbank and then imported into ChemBio3D Ultra 14.0 for energy minimization. The minimum root mean square (RMS) gradient was set to 0.001, and the small molecule was saved in “mol2” format. The optimized small molecule was imported into AutodockTools-1.5.6 for hydrogenation, charge calculation, and charge distribution, and then, the rotatable key was saved in “pdbqt” format. The FAST sequence of the target protein was downloaded from the Uniprot database, homologous modeling was performed using α-FOLD, and the protein crystal water and original ligand were removed from the modeled protein structure using “PyMOL2.3.0”. The protein structure was imported into “AutoDocktools” (v1.5.6) for hydrogenation, charge calculation, charge assignment, and atom type assignment, and saved in “pdbqt” format. POCASA 1.1 was used to predict the binding sites of compounds and target proteins, AutoDock Vina1.1.2 was used for docking with 10,000 compounds, and “PyMOL2.3.0” was used to analyze the interaction mode of the top three compounds with binding energy. The migration ability of CRC cells by inhibiting target proteins was evaluated after being treated with these three compounds.

### Statistical analysis

Data were presented as mean ± standard deviation. Significance of differences for the values was determined using the Student's *t*-test with Prism software (GraphPad Software, Inc., San Diego, California, USA). A *p*-value less than 0.05 was considered statistically significant.

## Results

### MFAP2 significantly up-regulated in CRC

**Figure 1:**
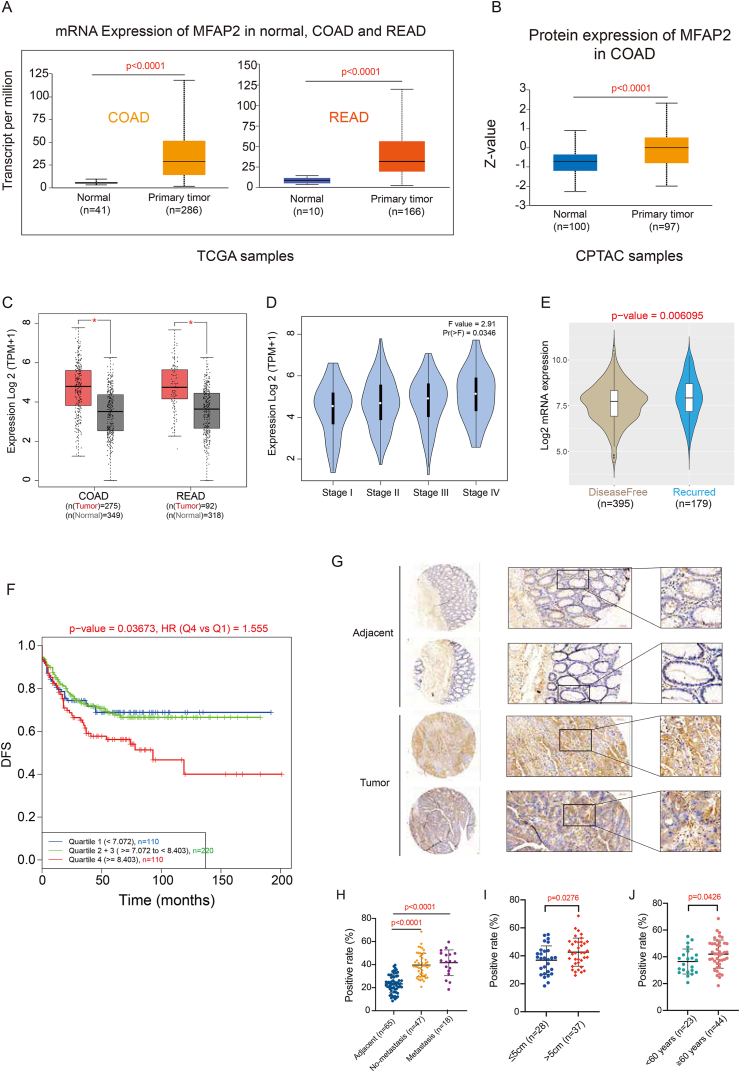
MFAP2 is identified as a potential prognostic biomarker for colorectal cancer (CRC). **(A, B)** The mRNA and protein expression of MFAP2 was significantly higher in colon adenocarcinoma (COAD) and rectal adenocarcinoma (READ), validated through various data sources, including TCGA (A) and CPTAC (B). **(C, D)** MFAP2 was significantly up-regulated in CRC patients and related to tumor progression, validated by GEPIA data. **(E)** Patients with tumor recurrence were accompanied by significantly higher expression of MFAP2. **(F)** CRC patients with high MFAP2 expression had significantly lower disease-free survival (DFS). **(G**–**J)** Immunohistochemical results showed that MFAP2 was significantly overexpressed in CRC tissues, and MFAP2 expression was higher in patients with larger tumor sizes or older age.

MFAP2 levels were significantly higher in tumor samples than in healthy tissues, according to an examination of samples from the TCGA and CPTAC databases for colon and rectal adenocarcinomas (Fig. 1A–C). Tumor growth and recurrence were linked to this rise in MFAP2 expression (Fig. 1D and E). Furthermore, the relationship between MFAP2 expression and disease-free survival was investigated using the CANCERTOOL database, which showed that patients with increased MFAP2 expression had a worse prognosis (Fig. 1F). These findings imply that MFAP2 might be involved in the course of CRC development. To confirm the aberrant expression of MFAP2 in CRC, immunohistochemical labeling and profile quantitative analysis were carried out on a CRC tissue array. A total of 65 matched cancer and surrounding tissues had their MFAP2 protein levels measured (Fig. S3A). The results showed that tumor samples had significantly greater levels of MFAP2 expression than nearby tissues (*p* < 0.0001) (Fig. 1G and H). Remarkably, compared to cancer tissues smaller than 5 cm, MFAP2 expression was substantially greater in those larger than 5 cm (Fig. 1I). The results for the groups aged ≥60 and ≥60 years were similar (Fig. 1J). These results imply that MFAP2 accelerates the development of colorectal cancer.

### MFAP2 knockdown impaired the proliferation and migration abilities of CRC cells

**Figure 2:**
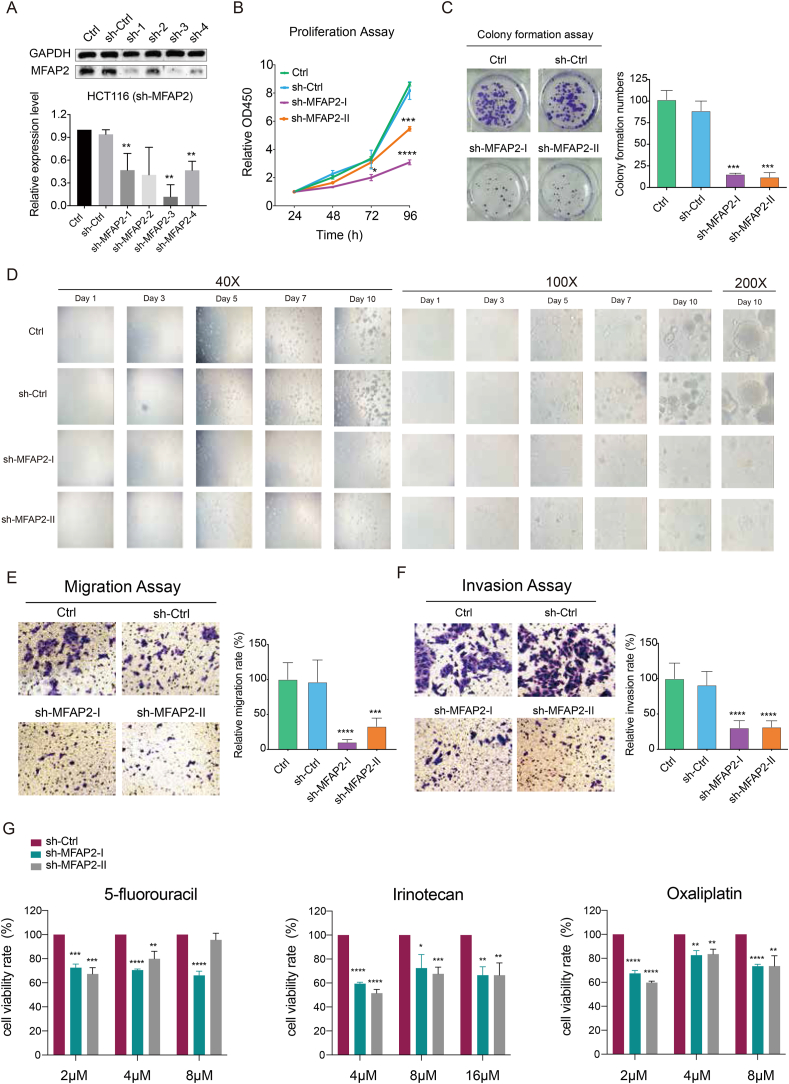
Functions of MFAP2 in colorectal cancer (CRC) cells. **(A)** The cell lines with stable MFAP2 knockdown were constructed, and the two groups (sh-MFAP2-3 and sh-MFAP2-4) with the lowest MFAP2 expression were selected for subsequent functional studies. **(B)** MFAP2 knockdown significantly reduced the proliferation of CRC cells. **(C)** MFAP2 knockdown significantly reduced the colony formation of CRC cells. **(D)** MFAP2 knockdown decreased the ability to form 3D tumor spheres. **(E, F)** MFAP2 knockdown significantly reduced the migration and invasion of CRC cells. **(G)** CRC cells with MFAP2 knockdown had lower viability rates when treated with the same concentration of 5-fluorouracil, irinotecan, or oxaliplatin than sh-Ctrl cells.

To explore the possible roles of MFAP2, four distinct CRC cell lines (HCT116, HT29, SW620, and HCT15) were employed. HCT116 cells were used as a cell model because they expressed more MFAP2 (Fig. S1). Initially, four distinct short hairpin RNAs (shRNAs, whose sequences are listed in Table S1) were used to establish an MFAP2-knockdown HCT116 cell line. Western blotting analysis was used to demonstrate MFAP2 knockdown (Fig. 2A). MFAP2-knockdown CRC cells were less able to proliferate than normal CRC cells, according to the CCK8 assay results (Fig. 2B). Additionally, it was noted that the size of 3D tumor spheres and the quantity of colonies were reduced (Fig. 2C and D; Fig. S2). These results suggest that MFAP2 play a crucial role in promoting the proliferation of CRC cells.

Transwell experiments were used to investigate the effect of MFAP2 on CRC cell metastasis. These findings demonstrated that the number of cells that migrated and invaded decreased when MFAP2 was reduced (Fig. 2E and F). It suggests that MFAP2 has a major impact on CRC metastasis. Furthermore, when subjected to the same concentration of chemotherapeutic agents (5-fluorouracil, irinotecan, and oxaliplatin), cells with MFAP2 knockdown showed a lower viability rate than normal CRC cells (Fig. 2G; Fig. S3). It is hypothesized that MFAP2 knockdown greatly amplifies the proliferation-inhibiting effect that 5-fluorouracil or oxaliplatin therapy has on CRC cells.

### MFAP2 regulated EMT function in CRC cells

**Figure 3:**
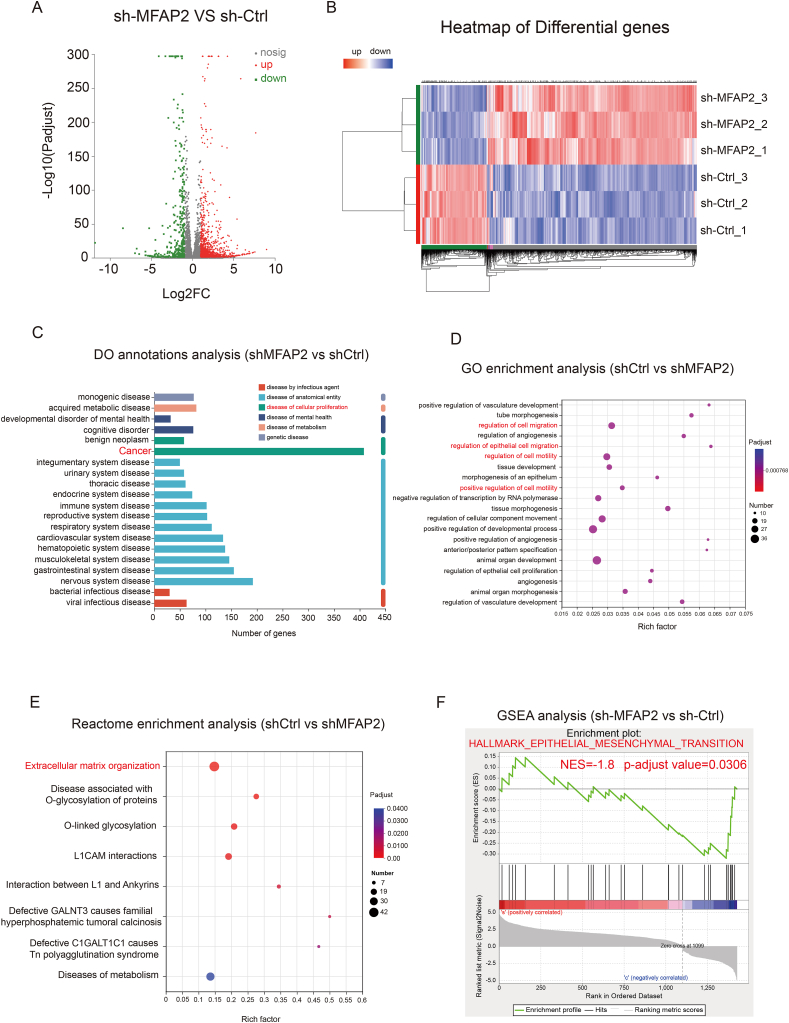
Transcription profiling analysis of colorectal cancer (CRC) with stable MFAP2 knockdown and sh-Ctrl cells. **(A, B)** Volcano map and heatmap of differentially expressed genes in the sh-MFAP2 and sh-Ctrl groups. **(C)** Disease Ontology (DO) annotation analysis revealed the types of diseases associated with differentially expressed genes, with the most genes associated with cancer. **(D)** Gene Ontology (GO) enrichment analysis showed that differentially expressed genes were involved in the regulation of cell migration and motility. **(E)** Reactome enrichment analysis suggests that MFAP2 knockdown mainly influences extracellular matrix organization. **(F)** Gene significantly enrichment analysis (GSEA) analysis showed that MFAP2 knockdown impacted epithelial–mesenchymal transition (EMT) in CRC.

The transcriptome data analysis revealed that between the sh-MFAP2 and sh-Ctrl groups, 397 genes were down-regulated and 1275 genes were up-regulated (Fig. 3A and B; Table S2). The majority of the differential genes were linked to cancer, according to the Disease Ontology (DO) annotations study (Fig. 3C). Furthermore, these differentially expressed genes may control cell migration and motility, according to the Gene Ontology (GO) enrichment analysis (Fig. 3D). These divergent genes were mainly engaged in extracellular matrix organization activities, according to a reactome enrichment analysis (Fig. 3E). Moreover, differentially expressed genes may be linked to EMT, according to GSEA analysis (Fig. 3F).

**Figure 4:**
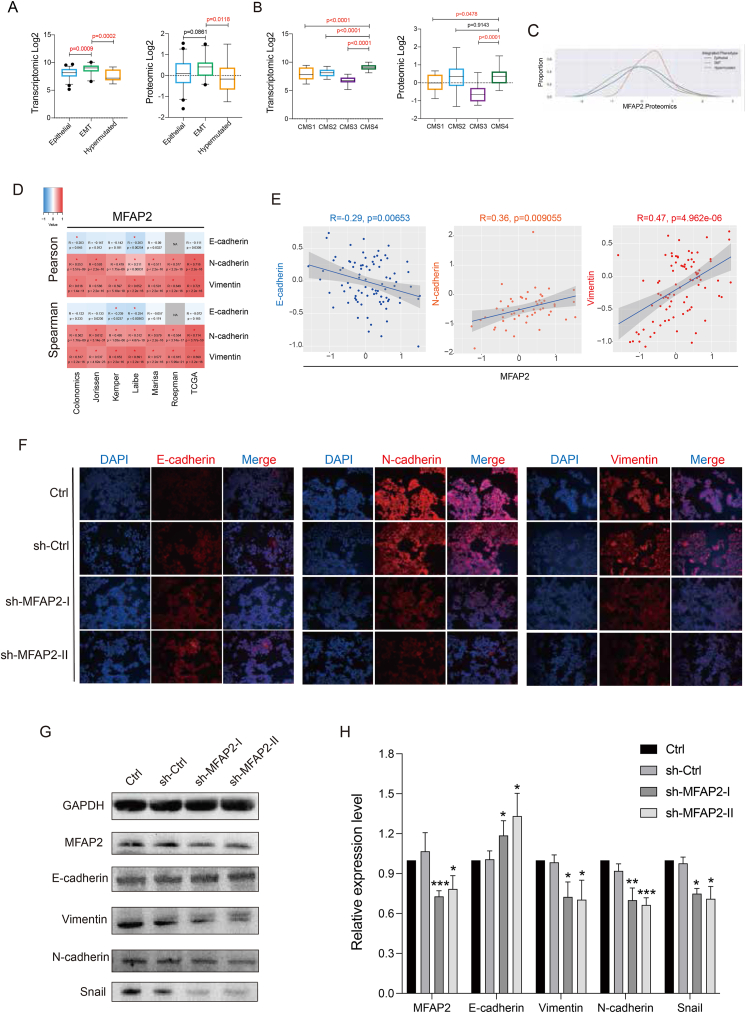
MFAP2 is associated with epithelial–mesenchymal transition (EMT) and regulates EMT through the TGF-β signaling pathway. **(A**–**C)** CPTAC transcriptomic and proteomic data showed that MFAP2 was highly expressed in EMT phenotype and CMS4 subtype patients. **(D, E)** Transcriptomic and proteomic data showed that MFAP2 expression was significantly negatively correlated with E-cadherin and positively correlated with N-cadherin and vimentin. **(F)** Immunofluorescence experiments further confirmed that the expression of MFAP2 was significantly negatively correlated with E-cadherin and positively correlated with N-cadherin and vimentin. **(G, H)** Western blotting showed that the decrease of MFAP2 expression could increase the expression of E-cadherin and decrease the expression of vimentin, N-cadherin, Snail, and TGF-β.

Additionally, MFAP2 was shown to be substantially expressed in EMT and CMS4 patients based on the analysis of transcriptome and proteomic data from CPTAC (Fig. 4A–C; Table S3–S6). MFAP2 had a strong negative correlation with E-cadherin (*r* = −0.29, *p* = 0.00653) and a substantial positive correlation with N-cadherin (*r* = 0.36, *p* = 0.009055) and vimentin (*r* = 0.47, *p* = 4.962e-06), according to the CANCERTOOL transcriptomic and CPTAC proteomic data (Fig. 4D and E; Table S12). The findings of the western blotting and immunofluorescence tests demonstrated that when MFAP2 expression was decreased in CRC cells, E-cadherin levels rose. Snail, vimentin, and N-cadherin levels, on the other hand, dropped. These proteins are essential regulatory factors in the development of EMT and play a significant role in its progression (Fig. 4F–H; Table S13). Overall, these results suggest that MFAP2 promotes tumor metastasis and drug resistance by regulating EMT-related processes.

### MFAP2 knockdown impaired EMT through the EGFR-AKT-STAT3 signaling pathway in CRC cells

**Figure 5:**
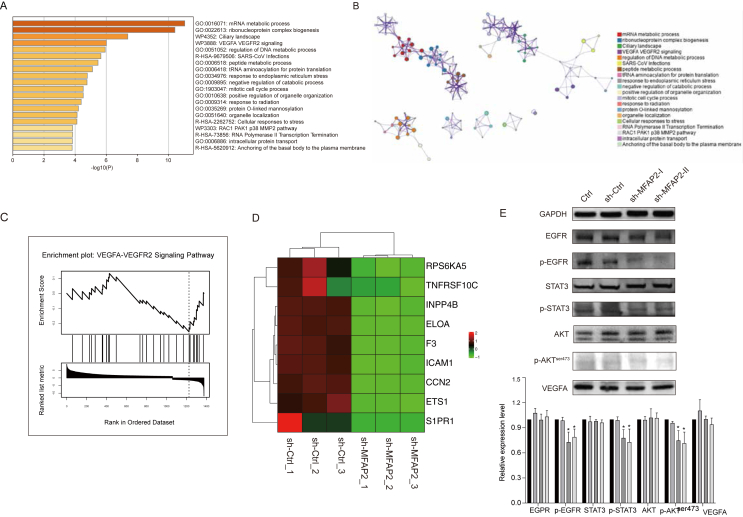
MFAP2 promotes epithelial–mesenchymal transition (EMT) through the EGFR-AKT-STAT3 signaling pathway in colorectal cancer (CRC) cells. **(A, B)** The top 20 enrichment signaling pathways regulated by MFAP2 knockdown. **(C, D)** VEGFR2 signaling pathway was enriched in the MFAP2 knockdown cells, shown by Gene Set Enrichment Analysis (GSEA) and essential genes in this enrichment. **(E)** MFAP2 knockdown affected the EGFR-AKT-STAT3 axis.

The links of significantly altered genes controlled by MFAP2 knockdown were shown by the Molecular Complex Detection (MCODE) network through full transcription profile analysis (Fig. 5A). The VEGFA-VEGFR2 signaling route was one of the top 20 enriched signaling pathways that were compiled (Fig. 5B). Furthermore, GESA revealed that the MFAP2-knockdown cells had a highly positive enrichment of the VEGFA-VEGFR2 signaling pathway (Fig. 5C). The VEGFA-VEGFR2 signaling pathway's key genes, such as RPS6KA5, CCN2, TNFRSF10C, INPP4B, ELOA, F3, ICAM1, ETS1, and S1PR1, were markedly altered in the MFAP2-knockdown cell lines (Fig. 5D). Furthermore, p-EGFR, p-STAT3, and p-AKT levels were reduced by MFAP2 knockdown (Ser473), while EGFR, STAT3, and AKT expression remained unaffected (Fig. 5E).

EGFR is essential for EMT. Through AKT, EGFR controls EMT-related proteins. By up-regulating E-Cadherin and down-regulating vimentin and Snail, EGFR knockdown may reduce prostate cancer's capacity to migrate and invade.^30^^,^^33^ When considered collectively, it was proposed that MFAP2 knockdown might impact CRC function by lowering EMT levels through the EGFR-AKT-STAT3 circuit.

### MFAP2 promoted the interaction between tumor cells and associated stromal cells

**Figure 6:**
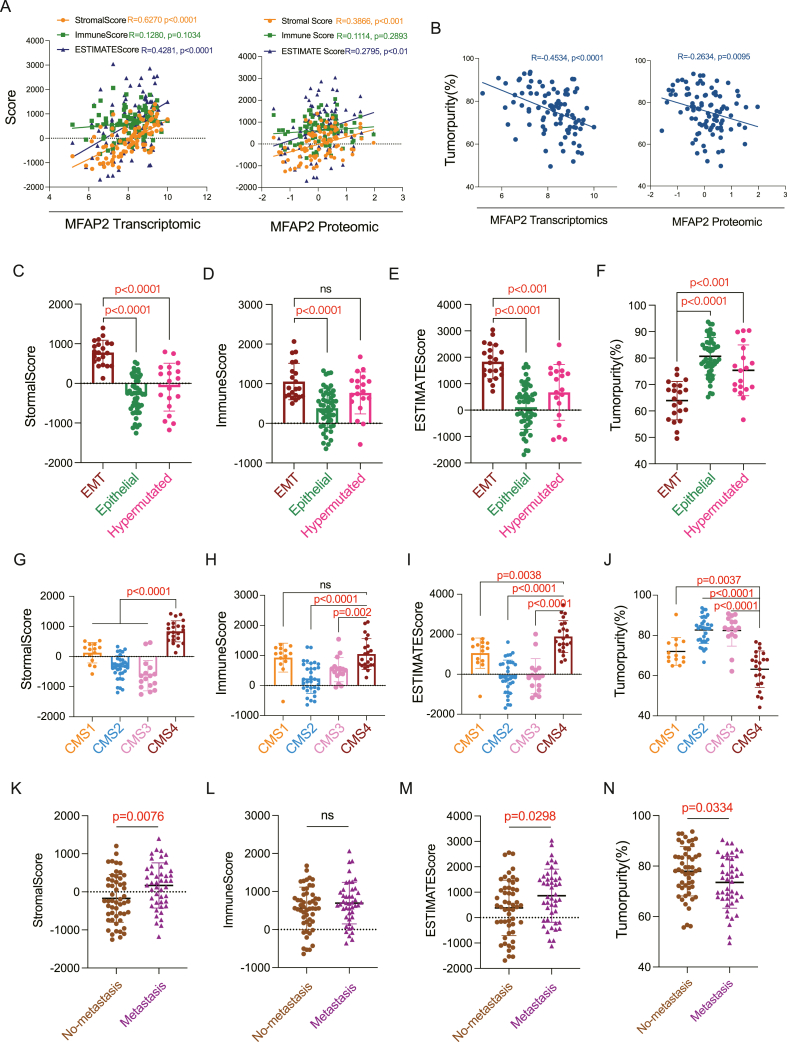
Tumor stromal score and tumor purity are significantly correlated with MFAP2 and epithelial–mesenchymal transition (EMT). **(A)** MFAP2 transcription and protein expression levels were positively correlated with tumor stromal scores. **(B)** MFAP2 transcription and protein expression levels were negatively correlated with tumor purity. **(C–F)** The tumor stromal score of the EMT phenotype was significantly higher than that of other cases, while the tumor purity was significantly lower than that of other cases. **(G**–**J)** The tumor stromal score of CMS4 subtype was significantly higher than that of other cases, while the tumor purity was significantly lower than that of other cases. **(K–N)** The tumor stromal score of metastatic CRC patients was significantly higher than that of other cases, while the tumor purity was significantly lower than that of other cases.

Based on biological process research, MFAP2 knockdown mostly affected tumor cell migration and motility as well as extracellular matrix architecture through EMT (Fig. 3D and E). The transcriptome and proteomic data from CPTAC were thoroughly examined in order to learn more about how MFAP2 controls EMT in CRC. According to the findings, MFAP2 expression levels were negatively connected with tumor purity (Fig. 6B) and positively connected with tumor stromal scores (Fig. 6A), which show a larger concentration of stromal cells in related tumor tissues.^34^ However, there was no significant correlation with immune scores (Fig. 6A).

The stromal and immunological scores are added to determine the ESTIMATE score. This study examined how EMT-type CRC cases differed from other types of cases in terms of tumor purity, stromal score, and immunological score. EMT phenotypes and CMS4 subtypes had much higher stromal scores than other kinds, although their tumor purity was significantly lower, according to the data (Fig. 6C–J; Table S9). Patients with metastases had significantly lower tumor purity and a significantly higher tumor stromal score than those without metastases (Fig. 6K–N). It was discovered that the immunological scores of EMT/CMS4 and hypermutated/CMS1 subjects did not differ significantly. This could be because hypermutated and CMS1 cases have substantial immune activation,^35^^,^^36^ which suggests that the increased contact between CRC cells and immune cells is not specific to EMT instances. These findings showed that a more useful metric for differentiating EMT-type cases among other CRC patient types would be the tumor stromal score. In clinical diagnosis, this score may also be used as a novel indicator to find patients with EMT characteristics. According to these results, MFAP2 may encourage tumor cells to engage with stromal cells linked to the tumor, which could lower tumor purity and raise the risk of tumor degeneration and metastasis.

### MFAP2 synergistically regulated EMT and promoted tumor metastasis

**Figure 7:**
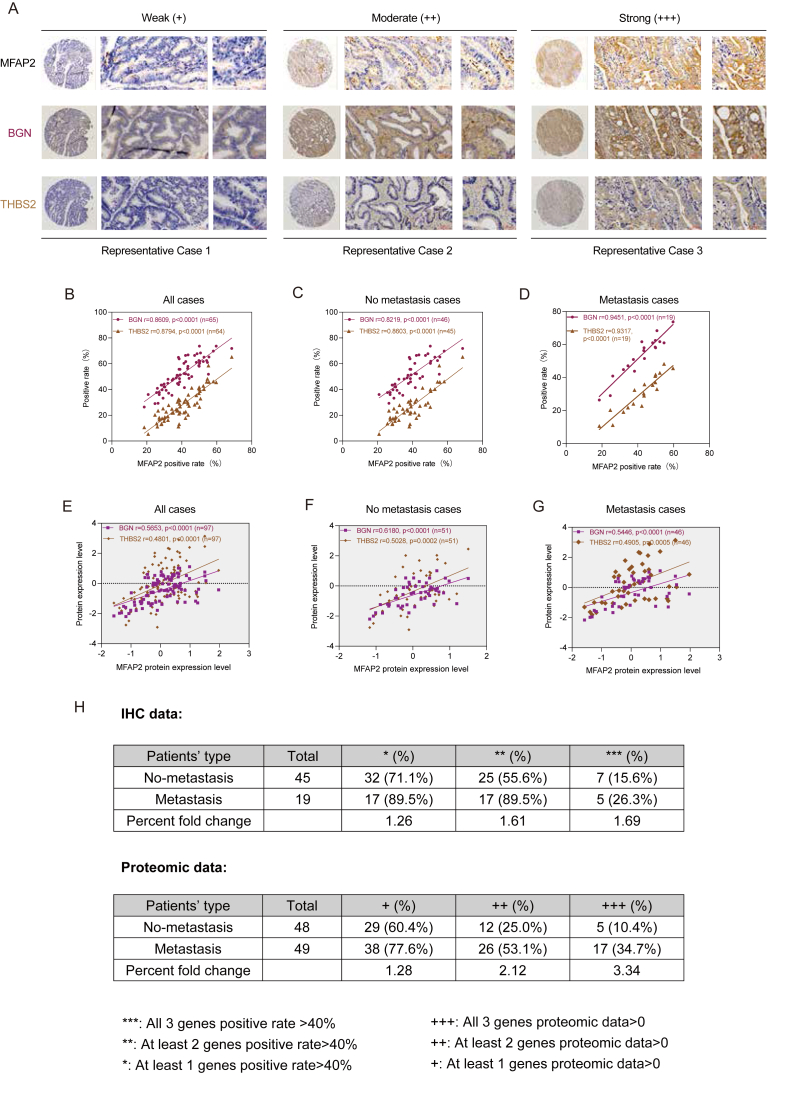
The diagnostic value of MFAP2, BGN, and THBS2 expression is significantly positively correlated in colorectal cancer (CRC) patients. **(A)** Immunohistochemical staining results of MFAP2, BGN, and THBS2 in CRC tissues of representative cases. **(B**–**D)** Immunohistochemical staining results showed that MFAP2 expression was significantly positively correlated with the expression of BGN and THBS2. **(E**–**G)** CPTAC proteomic data further confirmed that MFAP2 was significantly positively correlated with BGN and THBS2.

BGN and THBS2, which have been identified as important regulators that promote the metastasis of CRC cells by regulating EMT^28^ and are closely related to tumor stromal cells, such as cancer-associated fibroblasts,^37^, ^38^, ^39^ were correlated using tissue chip immunohistochemical and proteomic data. EMT and tumor metastasis have been linked to the BGN and THBS2^28^ (Fig. S3; Table S7). The research verified that typical EMT-related proteins were controlled by MFAP2. The positive rate of MFAP2 in CRC tissues was consistent with either BGN (*r* = 0.8609, *p* < 0.0001, *n* = 65) or THBS2 (*r* = 0.8794, *p* < 0.0001, *n* = 64), according to the immunohistochemistry data (Fig. 7A and B).

Both metastatic patients (MFAP2-BGN, *r* = 0.9451, *p* < 0.0001, *n* = 19; MFAP2-THBS2, *r* = 0.9317, *p* < 0.0001, *n* = 19) and non-metastatic cases (MFAP2-BGN, *r* = 0.8219, *p* < 0.0001, *n* = 46; MFAP2-THBS2, *r* = 0.8603, *p* < 0.0001, *n* = 45) showed the correlation (Fig. 7C and D). CPTAC proteomic data were used for further investigation in order to validate the association. According to the results, MFAP2 expression level and BGN/THBS2 were significantly positively correlated in all cases (MFAP2-BGN, *r* = 0.5653, *p* < 0.0001, *n* = 97; MFAP2-THBS2, *r* = 0.4801, *p* < 0.0001, *n* = 97), non-metastatic cases (MFAP2-BGN, *r* = 0.6180, *p* < 0.0001, *n* = 51; MFAP2-THBS2, *r* = 0.5028, *p* = 0.0002, *n* = 51), and metastatic cases (MFAP2-BGN, *r* = 0.5446, *p* < 0.0001, *n* = 46; MFAP2-THBS2, *r* = 0.4905, *p* = 0.0005, *n* = 46) (Fig. 7E–G; Table S8).

Higher expression levels of important regulatory proteins (MFAP2, BGN, and THBS2) were substantially linked to a higher likelihood of metastasis occurrence in CRC patients, as shown by the immunohistochemical and proteomic analyses (Fig. 7H). Additionally, transcriptome and proteomic data revealed that BGN and THBS2 expression levels were negatively connected with tumor purity (Fig. S4C, S4D, S4G, S4H) and positively associated with tumor stromal score (Fig. S4A, S4B, S4E, S4F, Table S11). When considered collectively, it is hypothesized that these EMT-regulating proteins are intimately associated with and crucial for CRC metastasis.

### MFAP2 identified as a potential druggable target for interfering with CRC metastasis

**Figure 8:**
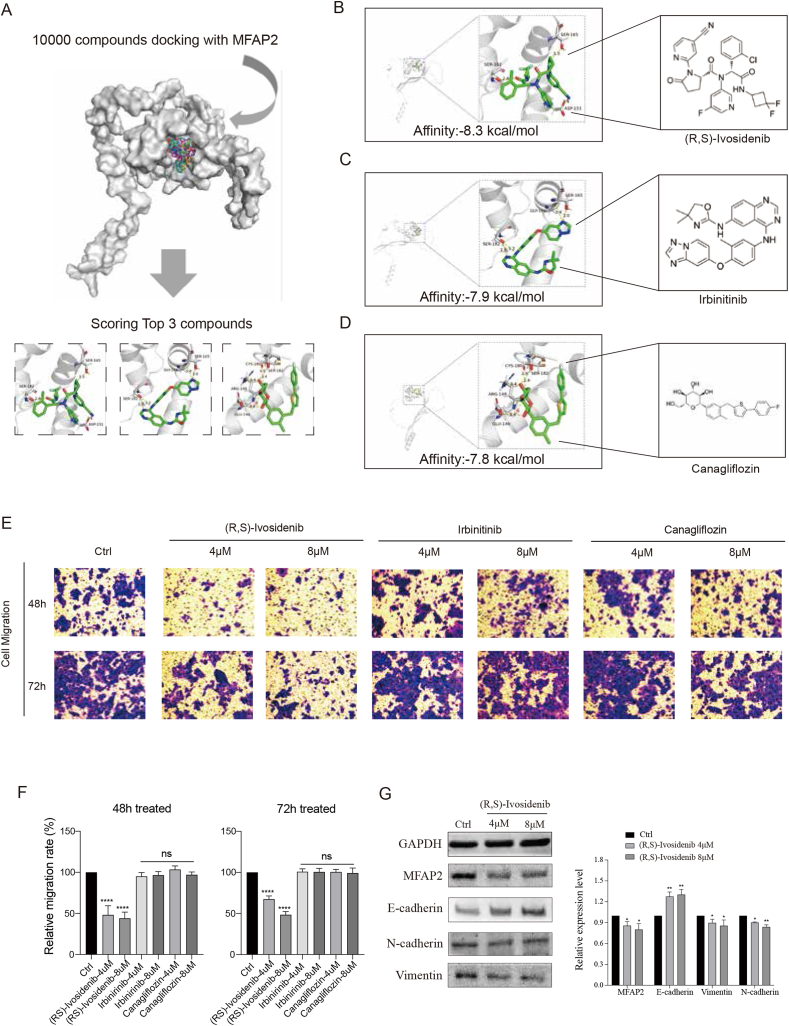
MFAP2 is identified as a potential therapeutic target for colorectal cancer (CRC). **(A)** Compounds with a strong binding force to MFAP2 were selected from 10,000 compounds by molecular docking. **(B**–**D)** (R, S)-ivosidenib, irbinitinib, and canagliflozin were the three compounds with the strongest binding force to MFAP2 among the 10,000 compounds. **(E, F)** (R, S)-ivosidenib significantly inhibited CRC cell migration at concentrations of 4 and 8 μM. **(G)** (R, S)-ivosidenib significantly inhibited MFAP2 expression and epithelial–mesenchymal transition (EMT)-related proteins at concentrations of 4 and 8 μM.

Molecular docking was conducted to explore the potential of MFAP2 as a druggable target for treating CRC metastasis. Ten thousand molecules were screened using molecular docking to find those that could attach to MFAP2 efficiently (Fig. 8A). According to the findings, canagliflozin, irbinirinib, and (R, S)-Ivosidenib had demonstrated potential in binding to MFAP2 (Fig. 8B–D). The therapeutic potential of MFAP2 as a possible target for CRC metastasis was initially investigated using these three drugs as tool molecules. Interestingly, at two higher concentrations (4 μM, 8 μM) (Fig. 8E, F), (R, S)-Ivosidenib strongly prevented the migration of CRC cells. According to the results of Western blotting, MFAP2, N-cadherin, and vimentin expression levels significantly decreased after receiving (R, S)-ivosidenib treatment.

In contrast, E-cadherin expression was markedly elevated (Fig. 8G). However, even at a dose of 8 μM, neither canagliflozin nor irbinirinib demonstrated a discernible inhibitory effect on migration. These findings imply that MFAP2 may be a viable therapeutic target for preventing CRC metastases; however, more investigation is required to pinpoint the precise mechanism.

## Discussion

In this study, MFAP2 was identified as a potential target for blocking CRC metastasis, which is significantly associated with EMT. A systematic evaluation of MFAP2's prognostic values and functions has been conducted. Both the transcriptional and protein levels of MFAP2 expression were much increased in CRC samples compared to normal samples. Furthermore, patients with higher MFAP2 expression levels fared worse in terms of survival than those with lower levels. To learn more about how MFAP2 acts in colorectal cancer, the stable MFAP2-knockdown CRC cell (HCT116) was created. MFAP2 knockdown prevented CRC cells from proliferating. Moreover, MFAP2 knockdown dramatically decreased CRC cells' capacity for invasion and migration. According to earlier research, MFAP2 can make CRC cells more aggressive, and MFAP2 expression inhibition can lessen the cancer's malignancy.^26^^,^^27^ Consistent with earlier research, our findings imply that MFAP2 may function as a regulator in the development of colorectal cancer (CRC) and may encourage the growth, advancement, and degradation of CRC in the presence of aberrant overexpression.

EMT is essential for development, and the mechanisms that underlie it are reactivated during fibrosis, cancer progression, and wound healing.^4^^,^^5^^,^^40^ In tumor tissues, EMT causes the mesenchymal phenotype, which is associated with treatment resistance and the migration, invasion, and metastasis of tumor cells.^41^ Studies that focus on EMT will offer fresh perspectives on how to manage tumor metastases. Improving the survival rate of patients requires preventing and treating tumor metastases. Previous research revealed that MFAP2 could promote melanoma cell invasion and migration via EMT and the Wnt/β-catenin pathway,^23^ enhance motility via the integrin α5β1/focal adhesion kinase (FAK)/extracellular signal-regulated kinase (ERK) pathway,^17^ and regulate EMT in gastric cancer through the transforming growth factor-beta (TGF-β) signaling pathway.^16^ However, little is understood about how MFAP2 controls metastasis and EMT in colorectal cancer. According to this study's transcriptome profile GESA analysis, MFAP2 may control the course of EMT and encourage the metastasis of CRC (Fig. 3F).

Patients with incurable colorectal cancer benefit from VEGF-inhibitory therapy because VEGF and its receptors are crucial for tumor growth and metastasis.^42^ Most patients with late-stage colorectal cancer have overexpressed EGFR, which is essential for carcinogenesis.^43^ In CRC cells, EGFR may activate downstream pathways like the ERK, AKT, and STAT3 pathways. Through the AKT and STAT3 signaling axis, VEGFR and EGFR may act independently of one another during oncogenesis and share downstream signaling pathways.^44^ Because VEGFR/EGFR plays a role in tumor start, angiogenesis, and metastasis, targeting it is appealing for CRC cancer therapy.^44^ Furthermore, EGFR controls EMT-related proteins via AKT, and by up-regulating E-cadherin and down-regulating vimentin and Snail, EGFR down-regulation may reduce prostate cancer's capacity to migrate and invade.^30^^,^^33^ According to this work, MFAP2 knockdown may have a similar pattern of significant effects on the expression of critical regulatory proteins related to EMT (Fig. 4G). Our investigation combined these hints to investigate the EGFR-related signaling route, although the findings showed that the VEGFA-VEGFR2 signaling pathway was more enriched (Fig. 5A and B). Additionally, EGFR (Tyr1068), STAT3 (Tyr705), and AKT (Ser473) phosphorylation levels may be dramatically decreased by MFAP2 knockdown, although EGFR, STAT3, and AKT expression levels did not alter significantly (Fig. 5E). Consequently, deactivated AKT may result from down-regulation of EGFR-related signaling following MFAP2 knockdown. It is highly probable that overexpressed MFAP2 controls aberrant CRC cell proliferation via the EGFR-AKT-STAT3 axis, which in turn controls CRC invasion, migration, and metastasis.

Cellular elements of the stroma cause EMT in tumor cells; the resulting mesenchymal tumor cells can react by altering the activities and representation of different cell types gathered in the stroma, particularly different immune cell subtypes that affect tumor growth.^45^, ^46^, ^47^ Our findings in this study showed that MFAP2 was intimately associated with tumor stromal cells (Table S10). It may also interact with EMT regulators to enhance tumor-stromal cell interaction and decrease tumor purity, hence establishing an environment that is conducive to the development and spread of tumors. Furthermore, there was a positive correlation between the expression level of MFAP2 and both BGN and THBS2, and a substantial positive correlation between the two variables and the tumor stromal score, but a negative correlation with tumor purity (Table S14). Higher levels of MFAP2, BGN, and THBS2 co-expression in tumor tissues were associated with a higher chance of metastasis in CRC patients. Thus, MFAP2 targeting may be a viable CRC therapeutic approach.

EMT can increase resistance to immunotherapy and chemotherapy by encouraging interaction with stromal cells associated with tumors.^6^^,^^7^^,^^9^ In the population of CRC patients, EGFR was overexpressed and strongly linked to the development of metastases in CRC patients.^43^ Twenty percent of patients with colorectal cancer had active phosphoinositide 3-kinase (PI3K)/AKT/mammalian target of rapamycin (mTOR) signaling axis, downstream signaling of EGFR, which would cause high expression of programmed death ligand 1 (PD-L1) in CRC stem cells and promote immune escape.^48^ Remarkably, TWIST1 activates MFAP2 expression to advance CRC stemness and oxaliplatin resistance.^49^ Our findings suggest that CRC metastases may be prevented by suppressing MFAP2 expression.

**Figure 9:**
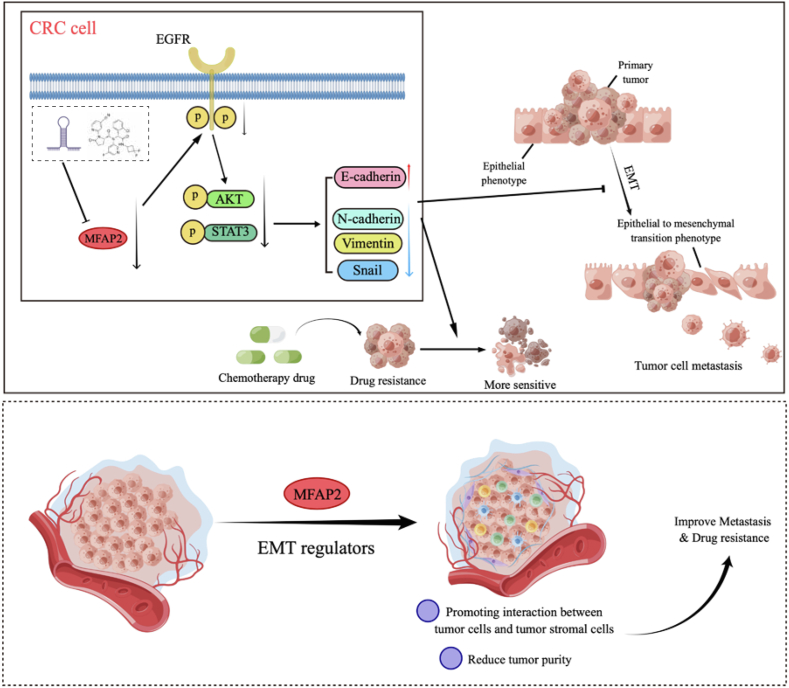
Proposed mechanism of MFAP2 promoting colorectal cancer (CRC) metastasis and drug resistance.

It has been proposed that decreased MFAP2 expression may increase CRC cells' susceptibility to chemotherapeutic treatments. Additionally, our findings showed that MFAP2 knockdown enhanced the inhibitory effect on CRC cell growth when 5-fluorouracil, oxaliplatin, or irinotecan—common first-line treatments for CRC—were administered.^50^ In order to improve the dismal outcomes of CRC patients who have metastasized, this suggests that screening for drugs that can block or suppress MFAP2 expression in CRC could be a helpful novel therapy method (Fig. 9).

In light of these results, MFAP2 was investigated further as a possible therapeutic target for colorectal cancer using molecular docking. Strong MFAP2 binding properties were found to be promising drug candidates for the treatment of colorectal cancer in clinical settings. Significantly, this study discovered that (R,S)-ivosidenib could inhibit MFAP2 to reduce EMT progression and successfully prevent CRC cell migration at particular concentrations. This is clinically applicable for the treatment of cholangiocarcinoma^53^^,^^54^ and relapsed/refractory mutant isocitrate dehydrogenase 1-positive (IDH1m+) acute myeloid leukemia.^51^^,^^52^ Consequently, our research indicates that ivosidenib has a promising clinical use in the treatment of colorectal cancer. These results suggested that MFAP2 might be a target protein for an EMT-based treatment approach for metastases of colorectal cancer.

In conclusion, this study raises the possibility that MFAP2 may serve as a new important regulator linked to EMT and a viable therapeutic target for reducing the spread of colorectal cancer. Through the EGFR signaling pathway, MFAP2 regulates EMT, which affects metastasis and treatment resistance in colorectal cancer. Additionally, tumor purity and tumor stromal score might become new standards for categorizing individuals with EMT-type colorectal cancer. It seems that MFAP2 is essential to the tumor microenvironment. Specifically, MFAP2 expression suppression or inhibition markedly reduced the migratory and invasive potential of CRC cells. Additionally, CMS4 CRC patients had significantly greater levels of MFAP2 expression than individuals with other subtypes. Interestingly, EMT appears to be hampered by MFAP2 suppression. All of these results suggest that, in comparison to other CRC subtypes, CMS4 patients may benefit more from a treatment approach that targets MFAP2.

## CRediT authorship contribution statement

**Zhicheng He:** Writing – original draft, Visualization, Validation, Investigation, Data curation, Conceptualization. **Yuanzhi Chen:** Methodology, Data curation. **Shuting Yang:** Validation, Methodology. **Cheng Chen:** Visualization, Validation, Investigation. **Yingying He:** Writing – review & editing, Writing – original draft, Visualization, Methodology, Conceptualization. **Shubai Liu:** Writing – review & editing, Writing – original draft, Project administration, Methodology, Investigation, Funding acquisition, Data curation, Conceptualization.

## Funding

This work was supported by the grant for outstanding talent from abroad by Chinese Academic of Science and Startup Support Funding (No. E0241211H1 to Shubai Liu) and State Key Laboratory of Phytochemistry and Natural Medicines, Kunming Institute of Botany, the Chinese Academy of Sciences, China (No. Y8677211K1, Y8690211Z1 to Shubai Liu).

## Conflict of interests

The authors declared no competing interests.
