# Supplementary figures and images for: MFAP2 promotes metastasis and drug resistance by regulating epithelial-to-mesenchymal transition through EGFR signaling pathway in colorectal cancer cells
Source: Genes Dis. 2025 Aug 12;13(3):101800. doi: 10.1016/j.gendis.2025.101800 (PMC12914112; doi:10.1016/j.gendis.2025.101800)

Fig.S1

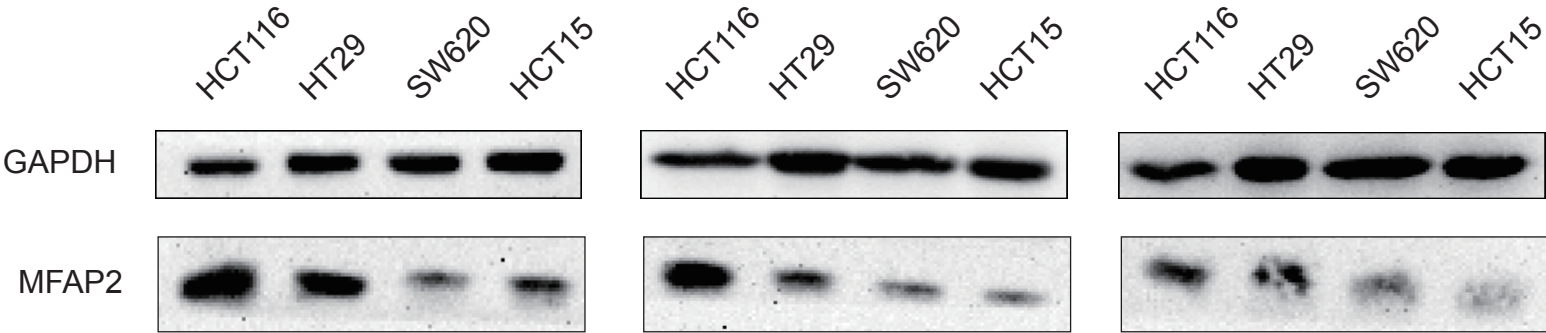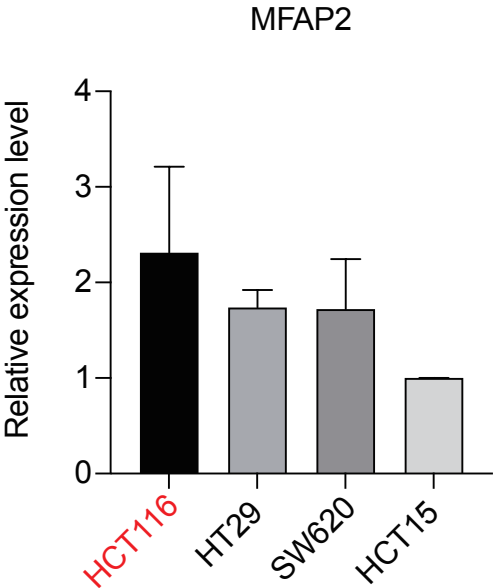

Fig.S2

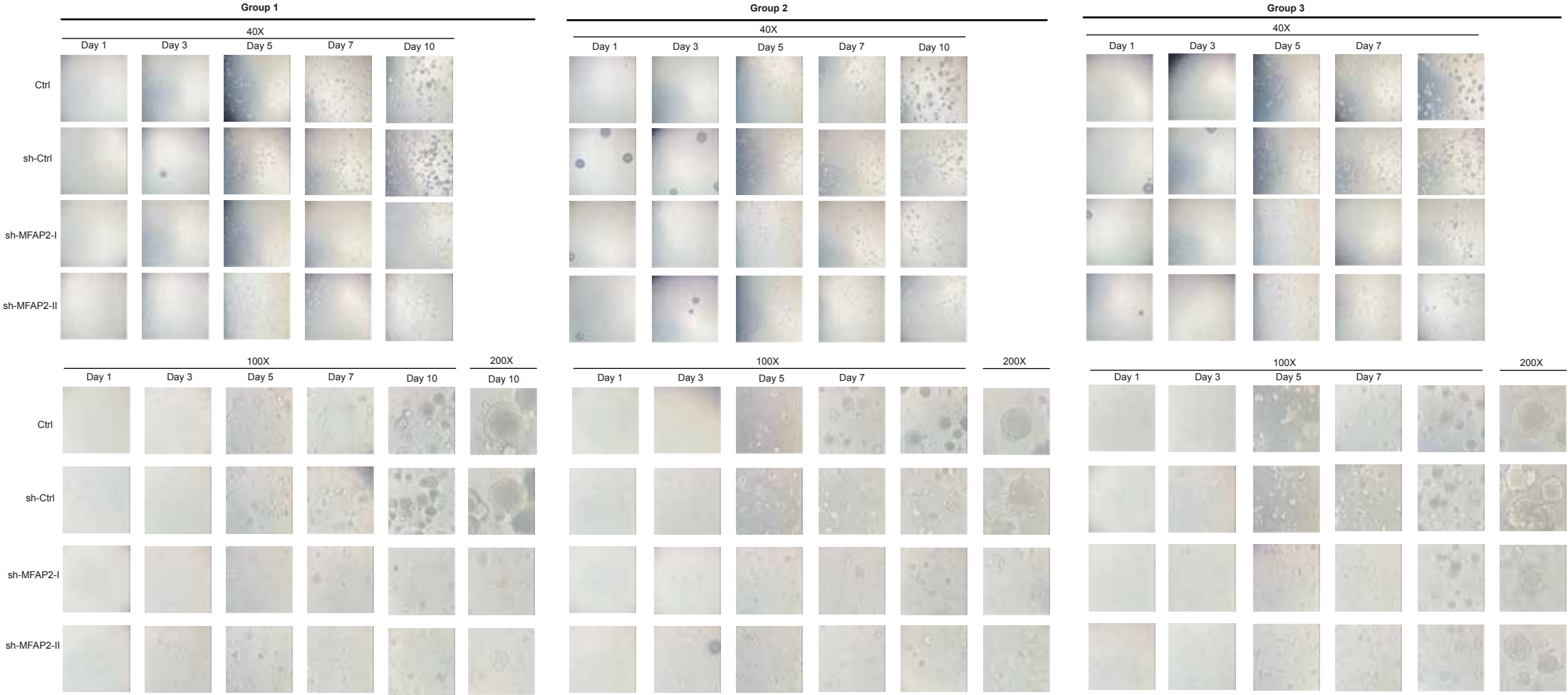

Fig.S3

A

BGN

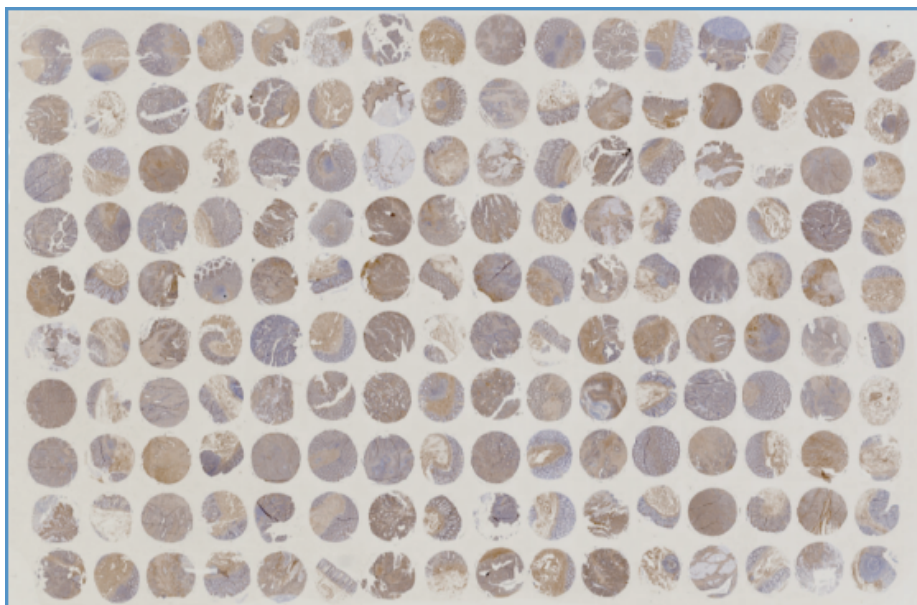

B

THBS2

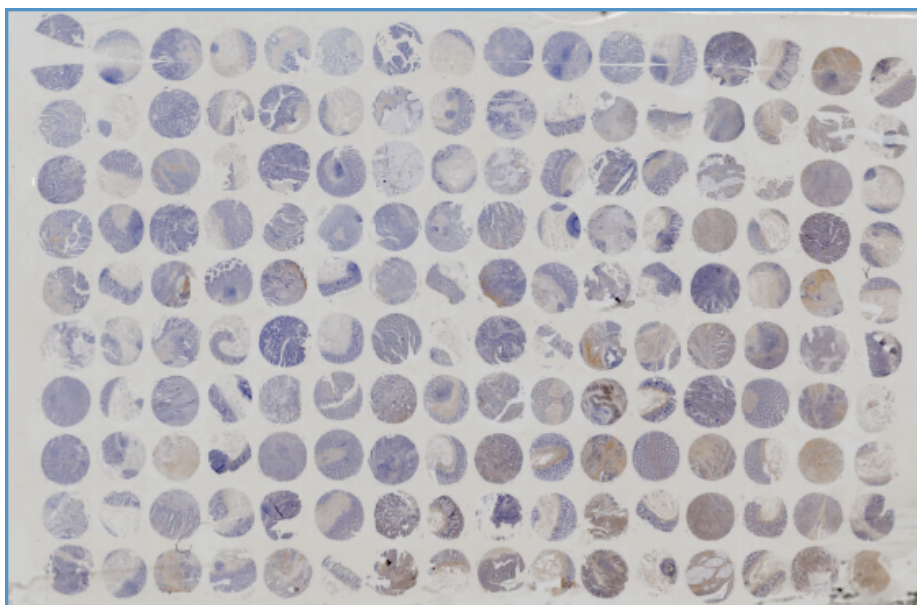

C

MFAP2

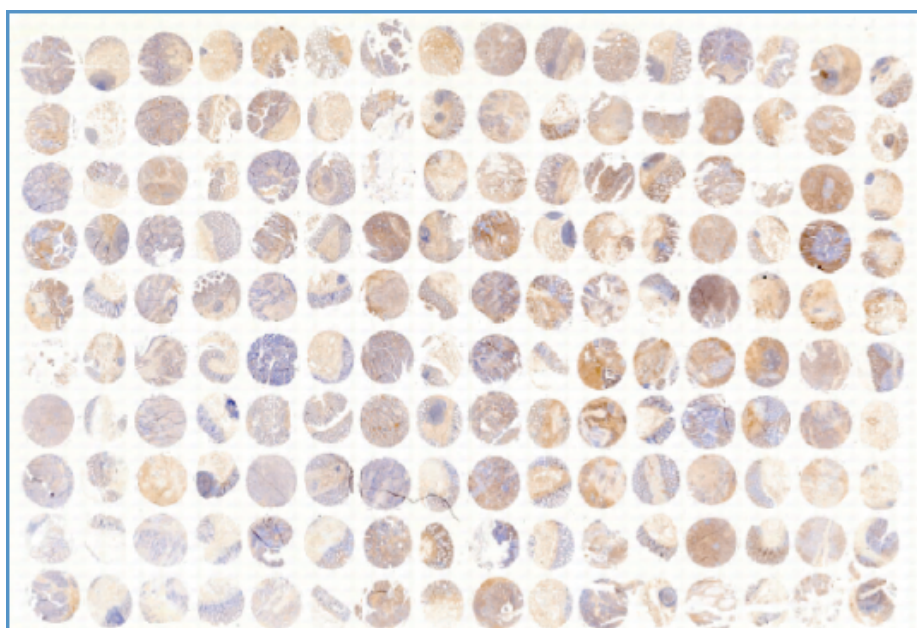

Fig.S4

A

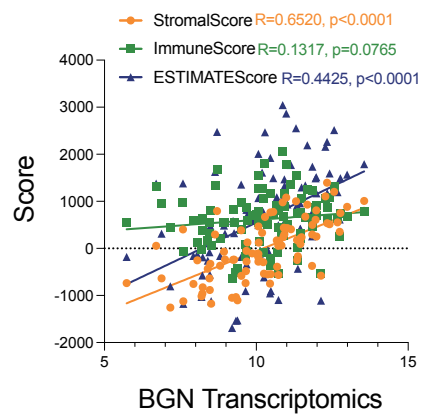

B

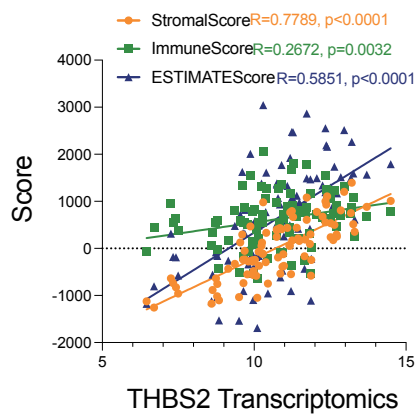

C

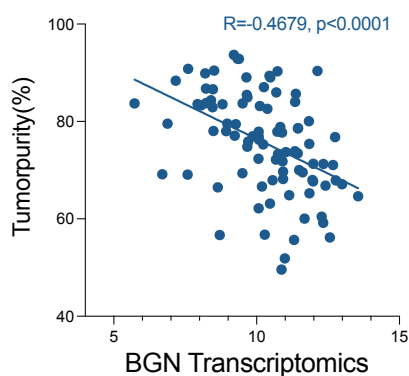

D

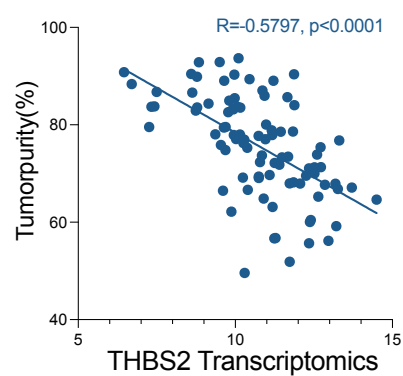

E

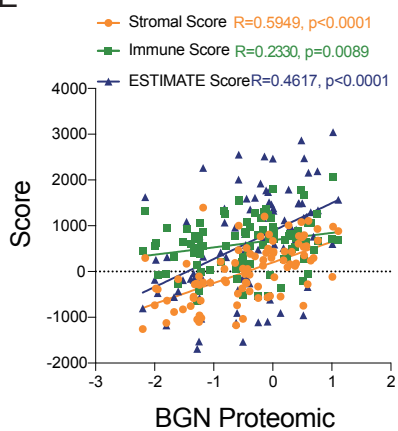

F

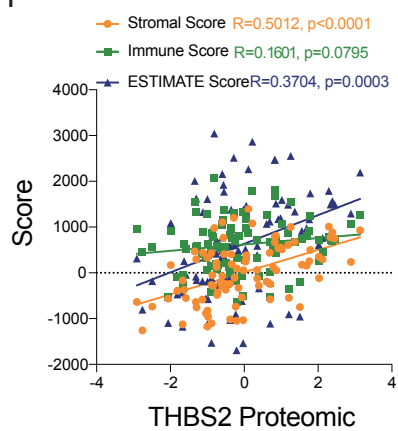

G

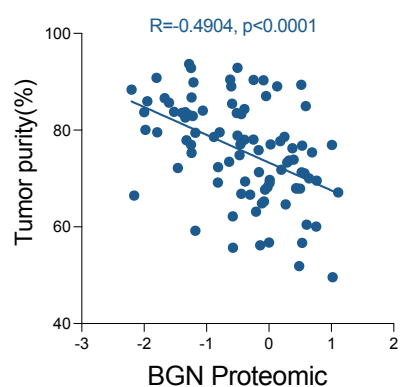

H

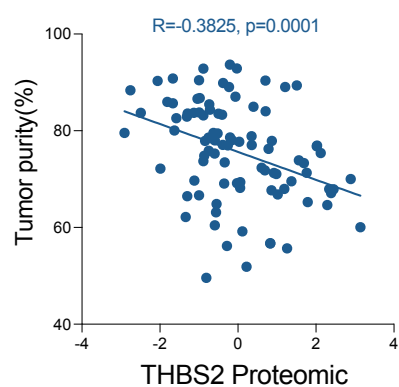

Supplement: Multimedia component 2 [file mmc2.pdf]
